# Cavity filling mutations at the thyroxine-binding site dramatically increase transthyretin stability and prevent its aggregation

**DOI:** 10.1038/srep44709

**Published:** 2017-03-24

**Authors:** Ricardo Sant’Anna, Maria Rosário Almeida, Nathalia Varejāo, Pablo Gallego, Sebastian Esperante, Priscila Ferreira, Alda Pereira-Henriques, Fernando L. Palhano, Mamede de Carvalho, Debora Foguel, David Reverter, Maria João Saraiva, Salvador Ventura

**Affiliations:** 1 Institut de Biotecnologia i Biomedicina and Departament de Bioquímica i Biologia Molecular, Universitat Autònoma de Barcelona, Bellaterra, Spain; 2 Instituto de Bioquímica Médica Leopoldo de Meis, Federal University of Rio de Janeiro, Rio de Janeiro, Brazil; 3 i3S – Instituto de Investigação e Inovação em Saúde da Universidade do Porto, Rua Alfredo Allen, 208, 4200 – 135 Porto, Portugal; 4 IBMC - Instituto de Biologia Molecular e Celular, Universidade do Porto, Rua Alfredo Allen, 208, 4200 – 135 Porto, Portugal; 5 ICBAS, Instituto de Ciências Biomédicas Abel Salazar, Universidade do Porto, Rua Jorge Viterbo Ferreira 228, 4050 – 313 Porto, Portugal; 6 Institute of Physiology-Instituto de Medicina Molecular, Faculty of Medicine, University of Lisbon, Lisbon, Portugal; 7 Department Neurosciences, Hospital de Santa Maria-CHLN, Lisbon, Portugal

## Abstract

More than a hundred different Transthyretin (TTR) mutations are associated with fatal systemic amyloidoses. They destabilize the protein tetrameric structure and promote the extracellular deposition of TTR as pathological amyloid fibrils. So far, only mutations R104H and T119M have been shown to stabilize significantly TTR, acting as disease suppressors. We describe a novel A108V non-pathogenic mutation found in a Portuguese subject. This variant is more stable than wild type TTR both *in vitro* and in human plasma, a feature that prevents its aggregation. The crystal structure of A108V reveals that this stabilization comes from novel intra and inter subunit contacts involving the thyroxine (T_4_) binding site. Exploiting this observation, we engineered a A108I mutation that fills the T_4_ binding cavity, as evidenced in the crystal structure. This synthetic protein becomes one of the most stable TTR variants described so far, with potential application in gene and protein replacement therapies.

Protein misfolding and aggregation into amyloid deposits is associated with the onset of an increasing number of human degenerative disorders^1^. Transthyretin (TTR) amyloidoses are diseases characterised by the extracellular deposition of fibrillar material containing TTR^2^. The wild-type protein (TTR WT) forms the amyloid deposits causing senile systemic amyloidosis (SSA)^3^, whereas the rest of TTR amyloidoses are caused by point mutations in the TTR primary sequence that exacerbate the intrinsic propensity of the protein to aggregate. TTR is a highly polymorphic protein, with more than 100 different point mutations associated with autosomal dominant hereditary amyloidosis, mainly familial amyloidotic polyneuropathy (FAP)^4^,^5^ and familial amyloid cardiomyopathy (FAC)^6^,^7^.

TTR is a homotetrameric plasma protein that carries L-thyroxine (T_4_)^8^ and vitamin A^9^,^10^. The TTR molecule is composed of four identical 127 amino acid subunits, named A, B, C and D. Each monomer consists of eight strands in a β-pleated sheet conformation. The TTR tetramer is formed by association of the AB and CD dimers. The weaker dimer-dimer interface defines two, largely unoccupied, funnel-shaped T_4_-binding sites^11^,^12^.

Pathogenic mutations affect the native TTR stability, facilitating the dissociation of the tetramer into monomers that, upon partial unfolding, aggregate into amyloid fibrils. Tetramer dissociation is the rate-limiting step for TTR aggregation. Accordingly, a synthetic variant with dimers formed by covalently bonded monomers could not form amyloid fibrils^13^,^14^. The fibrillation tendency of a given TTR variant and its stability are interconnected. Indeed, the disease severity, as defined by the age of onset and the penetrance of the pathology, can be predicted evaluating the *in vitro* thermodynamic and kinetic stability of the causing protein variant^15^,^16^. The crystal structures of TTR amyloidogenic variants have shown that these proteins usually exhibit altered contacts between dimers, that would account for the destabilization of their quaternary structure^17^,^18^,^19^,^20^.

For many years, liver or combined liver and heart transplantation were the only available treatments for the TTR amyloidoses^21^. Despite strong evidence linking TTR aggregation to the onset of the disease, it is not completely clear whether they are the initial soluble assemblies or the insoluble fibrils that exert the cytotoxic effect^22^,^23^. Therefore, prevention of the entire amyloidogenesis process appears as the most conservative therapeutic strategy for TTR diseases. This has been accomplished using small molecules able to bind to the TTR T_4_-binding sites, acting as kinetic stabilizers. They connect the hydrophobic surfaces of the AC and BD dimers through non covalent interactions, mainly of hydrophobic character, increasing the energy barrier of tetramer dissociation and stalling TTR aggregation^24^,^25^,^26^,^27^.

A reduced number of non-pathogenic mutations have been described for TTR and, so far, only two of them have been shown to be more stable than TTR WT. TTR T119M is a variant identified in the Portuguese population, which is present in plasma at higher levels than TTR WT due to a slower clearance of this mutant protein from serum^28^,^29^. Importantly, individuals carrying the T119M mutation together with the FAP associated V30M one, present a more benign evolution of the disease than heterozygote kindred carrying the V30M mutation alone^30^,^31^. Thus, this mutation acts as an inter-allelic trans-suppressor. Similar effects have been described for the variant TTR R104H found in heterozygote individuals from a Japanese family with FAP^32^.

The TTR T_4_-binding channels have three symmetrical sets of small depressions, termed halogen binding pockets (HBP), into which the four iodine atoms of the ligand are placed. The innermost binding pocket, HBP-3, is located between the side chains of Ser 117, Thr 119, Ala 108 and Leu 110. Similar to kinetic stabilizers, the protective effect T119M mutation is caused by the higher hydrophobicity it introduces in the channel at the interface between dimers^33^. This increases the mutant resistance to dissociation. Indeed, incorporation of one subunit of TTR T119M into a predominantly TTR V30M tetramer suffices to stabilize the mixed tetramer against dissociation^34^,^35^. In addition to the T119M mutation, the crucial role played by hydrophobicity at the T_4_-binding cavity on the stability of the TTR tetramer is exemplified by the non-natural L110A mutation. The HBP-2 binding pocket is formed by the side chains of Leu 110, Ala 108, Ala 109 and Leu 17. Decreasing HBP-3/HBP-2 hydrophobicity by shearing the Leu 110 side chain into an Ala promotes the fast dissociation of the tetramer into monomers^14^.

Here, we identified a novel A108V non-pathogenic mutation in a Portuguese subject. Because the side chain of Ala 108 is involved in the three TTR HBP sites, we speculated that this mutation would impact the protein stability. The crystal structure of this mutant and the characterization of its stability *in vitro* and in human plasma confirms TTR A108V as the third described natural variant displaying increased stability relative to TTR WT, being more stable *in vitro* than TTR T119M. As expected, TTR A108V stability precludes its aggregation into amyloid fibrils. We engineered a novel TTR A108I variant to attain an optimal packing of the hydrophobic residues in the T_4_-binding cavity, as confirmed in the crystal structure. This designed protein becomes one of the most stable TTR variants described so far, at the cost of being unable to bind the T_4_ hormone. Taken as a whole, the present study provides important novel insights on the molecular determinants accounting for TTR stability.

## Results

### Detection of a stable TTR A108V variant on a Portuguese Subject

Plasma TTR was immunoprecipitated with an anti-human TTR antibody and TTR variants identified by MALDI (matrix-assisted laser-desorption ionization) mass spectrometry analysis. The resulting peptide masses lists were compared with the theoretical tryptic peptide masses lists. In addition to a peptide encompassing residues 105–127 with 2,489 Da mass, corresponding to TTR WT, an abnormal peptide with a mass of 2,517 Da was detected (not shown). This change is compatible with an Ala to Val substitution in this region of the TTR sequence. Thus, the propositus carried both TTR WT and a mutated variant. We confirmed the identity and position of the mutation by DNA analyses. Sequence analysis of amplified TTR exon 4 revealed a C-to-T transition in the second base of codon 108, normally encoding an Ala, giving rise to a Val residue, in agreement with the mass spectrometry data.

**Figure 1:**
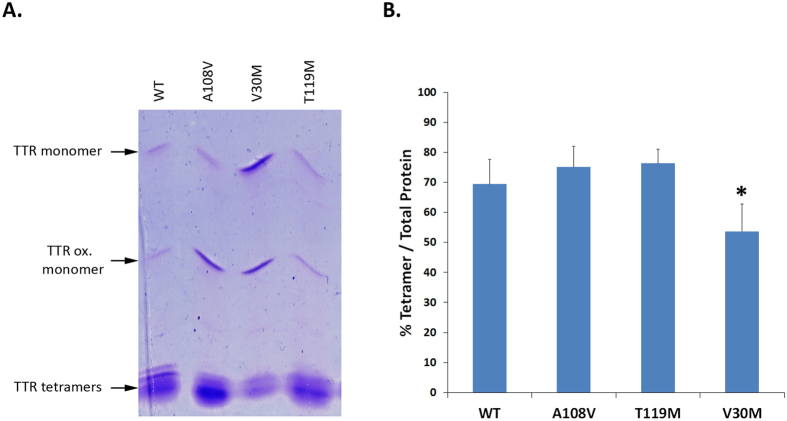
Assay of TTR tetrameric stability by IEF. (**A**) Plasma TTR from heterozygote carriers of TTR variants or from controls were isolated by native PAGE electrophoresis and then separated by IEF in semi-dissociating conditions (4 M urea). This is a representative picture selected from the 3 replicas considered for the quantification presented in (**B**). (**B**) Relative abundance of tetramer was calculated based on the ratio of intensity of tetramer bands (% Tetramer) over intensity of all TTR bands (Total TTR) in samples from plasma. *P < 0.05 (Student’s t-test). Error bars indicate SD.

Ala 108 maps into the TTR T_4_-binding channel. Despite the subject did not exhibit any evident symptom that could be associated with TTR amyloidosis, because mutations in this cavity have been shown to exert a strong impact on the tetramer stability, we decided to analyse the global TTR stability in the plasma of this person using native PAGE and Isoelectric Focusing (IEF) under semi-denaturing conditions (4 M Urea)^24^ (Fig. 1). Plasmas from heterozygote carriers of the V30M and T119M mutations were also included in the analysis, as references for variants with decreased and increased stability, respectively^29^,^36^, as well as control plasma. The presence of TTR monomer (M), oxidized monomer (Ox M) and several bands of lower pI corresponding to tetramers could be discriminated (Fig. 1). The results evidenced that in the TTR A108V heterozygote carrier there was a higher percentage of tetramer over total TTR (75.8 ± 6.8% tetramer) than in WT control plasma (69.4 ± 8.3% tetramer) (Fig. 1A and B), thus suggesting that the TTR A108V variant is more stable than the WT protein. The percentage of tetramer in TTR A108V heterozygote carrier plasma was similar to that found in TTR T119M heterozygote carrier plasma (76.3 ± 4.7% tetramer) and higher than that of the TTR V30M heterozygote carrier plasma (53.6 ± 9% tetramer).

### Computational prediction of TTR variants stability

We have shown recently that the impact of mutations on the TTR stability can be addressed computationally using the all-atom FoldX force field^37^. Although FoldX does not rearrange Ca upon mutation, the crystallography structures of most TTR mutants solved to date show no significant backbone deviation, relative to TTR WT. Therefore, we used FoldX to create a structural model for TTR A108V by using the TTR WT crystallographic structure (PDB: 1F41) as a template. From this model, the energies associated with the interactions gained or lost after mutation were quantified and used to predict the change in the structure thermodynamic stability (ΔΔG = ΔG_mut_ − ΔG_wt_). In good agreement with the measured stability in plasma, TTR A108V was predicted to be 8.30 kcal.mol^−1^ more stable than the WT protein. This over-stabilization seemed to result from the projection of the additional methylene group of Val into the T_4_ binding channel at the tetrameric interface (Fig. S1). Because the filling of the cavity with additional hydrophobic chemical groups has been reported to increase TTR stability^33^, we decided to test whether introducing four additional methylene groups by mutating Ala 108 into Ile would result in a denser packing of the T_4_-binding channel and, accordingly, in an over-stabilized protein variant. The modelled TTR A108I variant was predicted to be more stable than A108V by FoldX, with a ΔΔG of 13.71 kcal.mol^−1^ relative to TTR WT.

### Probing the thermodynamic stability of TTR A108V and TTR A108I variants *in vitro*

**Figure 2:**
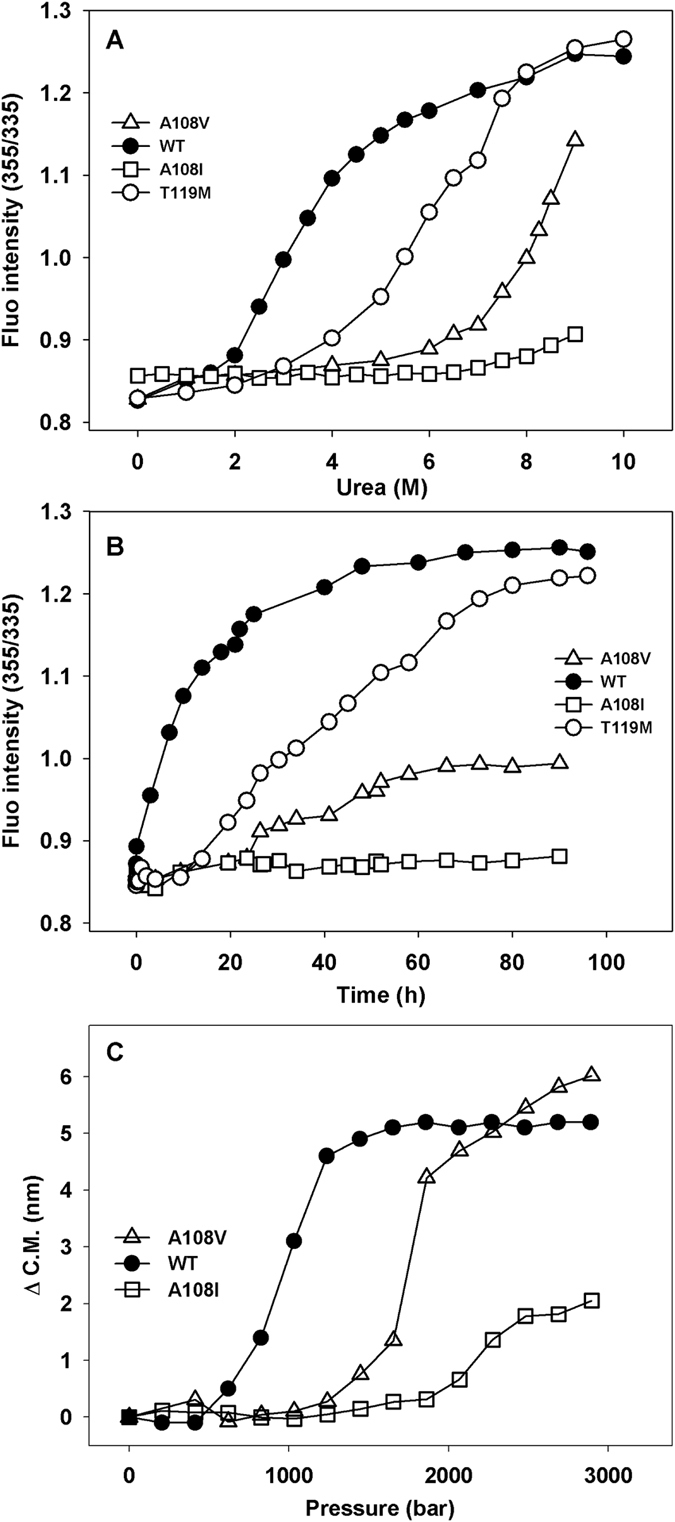
Assessing Thermodynamic (**A**) and kinetic (**B**) stabilities of TTR mutants by urea unfolding. In (**A**), samples were incubated at 1.8 μM at different urea concentrations for 96 hours. Excitation was set to 280 nm while emission of fluorescence scanned from 300 to 400 nm. In (**B**) samples were incubated at 8 M urea and denaturation followed along time. The ratio in intensity between 355 and 335 nm was used to measure the extent of denaturation. Panel (C) shows the denaturation profile of the three variants probed by HHP. Samples were incubated at 1.8 μM and HHP increased. Also Trp fluorescence was used as a probe of denaturation.

In order to validate experimentally the increase in stability predicted for TTR A108V and TTR A108I, both proteins were produced recombinantly, together with TTR WT and TTR T119M. The stabilities of all these variants were measured using equilibrium urea denaturation. The proteins were incubated for 96 h at increasing urea concentrations and changes in intrinsic Trp fluorescence monitored to calculate the percentage of folded protein at any given urea concentration (Fig. 2A). TTR denaturation by urea requires prior dissociation of the tetramer and, therefore, it indirectly reports on the protein kinetic stability. TTR WT begins to unfold at ∼2 M urea exhibiting the expected denaturation curve with a C_m_ = 3.2 M. As previously reported, TTR T119M is more stable than TTR WT, beginning to unfold at ∼3 M urea and displaying a C_m_ = 6.0 M. To our surprise, TTR A108V is much more stable, beginning to unfold only at ∼6 M urea and not being completely denatured at concentrations as high as 9 M urea. The stabilization promoted by the A108I mutation is even more dramatic, since this variant begins to unfold only above 8 M urea and its tetrameric structure seems to be essentially intact even at 9 M of the chaotropic agent.

Using urea concentrations in the posttransition region directly links the slow TTR quaternary structural changes to the rapid tertiary structural changes and renders unfolding irreversible, allowing to measure the kinetics of tetramer dissociation. The four proteins were incubated in 8 M urea and changes in Trp intrinsic fluorescence monitored along time (Fig. 2B). The t_1/2_ for dissociation of TTR WT is 5.5 h, whereas, as expected, TTR T119M dissociates slower, with a t_1/2_ of 42 h. From the experiment, it is clear that, at 8 M urea, both TTR WT and TTR T119M dissociate to a larger extent than TTR A108V and specially than TTR A108I, both mutants displaying extremely slow dissociation rates. This indicates that they are endorsed with a significantly increased kinetic stability, which will preclude tetramer dissociation on a biologically relevant time scale. Finaly, the stability of TTR A108V and A108I was probed by high hydrostatic pressure (HHP) by following the red shift in Trp emission maximum caused by the exposition of this residue to the aqueous environment when native TTR dissociates and unfolds. As shown in Fig. 2C, the sequence of thermodynamic stability recapitulates what has been seen with urea, namely, an enhanced stability of both varints when compared to the WT sequence, being TTR A108I pressure resistant, displaying only partial denaturation under HHP at 1 °C. The p_1/2_ values (pressure that furnishes 50% dissociation-denaturation) obtained for the dissociation-denaturation of TTR WT and A108V were equal to 974 bar and 1,755 bar.

**Figure 3:**
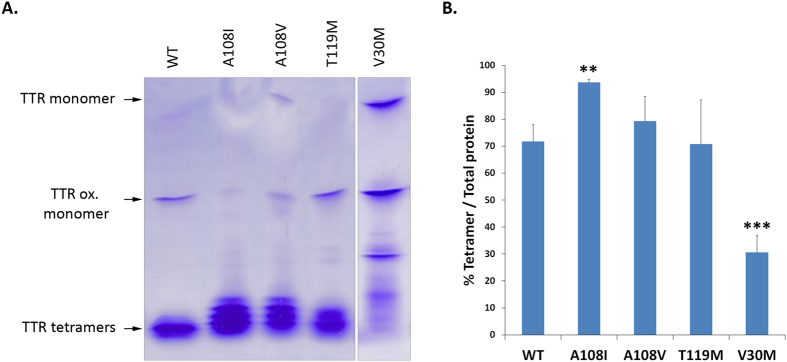
Assay of recombinant TTR A108V and TTR A108I tetrameric stability by IEF. (**A**) Recombinant TTR variants were isolated by native PAGE electrophoresis and then separated by IEF in semi-dissociating conditions (4 M urea). All samples were run in the same gel, but in the case of TTR V30M not side by side. This is a representative picture selected from the 3 replicas considered for the quantification presented in (**B**). (**B**) Relative abundance of tetramer was calculated based on the ratio of intensity of tetramer bands (% Tetramer) over intensity of all TTR bands (Total TTR) in samples from recombinant proteins. **P < 0.01; ***P < 0.001 (Student’s t-test). Error bars indicate SD.

We also assayed the tetramer stability of the purified recombinant proteins by IEF under semi-dissociating conditions (Fig. 3). In good agreement with equilibrium and kinetic data, the results demonstrated that the percentage of tetramer over total TTR was higher for TTR A108V (79.3 ± 9.2% tetramer) variant and for TTR A108I (93.7 ± 1.2%) than for TTR WT (71.7 ± 6.3%) and TTR V30M (30.6 ± 6.4%), but also higher than for the well-characterized TTR T119M trans-suppressor (70.8 ± 16.3%).

### Aggregation of TTR A108V and TTR A108I variants

**Figure 4:**
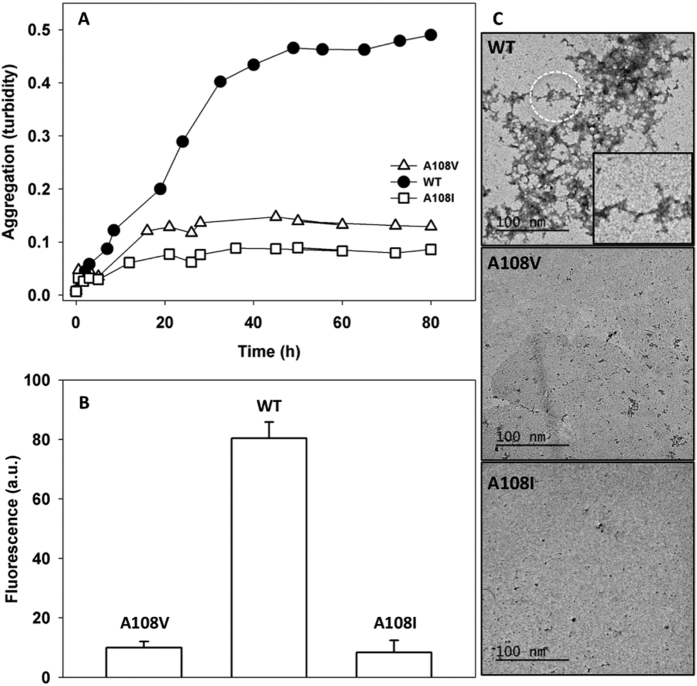
Aggregation: Samples were incubated at 3.5 μm at pH 4,4 and 37 °C with no agitation. Turbidity at 340 nm was monitored along time to measure aggregation (**A**). At the end of the experiment, a Th-T assay was performed to confirm the amyloid character of the aggregation (**B**). Exciting the samples at 450 and collecting the emission from 460 to 600 nm we obtained Th-T spectra. (**C**) shows TEM of the end point of the aggregated samples. Scale bars values are indicated.

The aggregation of TTR into amyloid fibrils is preceded by tetramer dissociation and the rate at which the tetramer dissociates to the amyloidogenic intermediate governs the rate of fibril formation. Therefore, it was expected that the increased kinetic stability of TTR A108V and TTR A108I may somehow reduce their aggregation, relative to TTR WT. In order to confirm this possibility, we assessed the kinetics of aggregation after incubating the proteins at pH 4,4 and 37 °C, a condition that induces partial denaturation and TTR amyloidogenesis. To this aim, we monitored the increase in solution turbidity along time (Fig. 4A). Despite the clinical and pathological history of domino transplants indicate that the seeding and nucleation are important elements of TTR amyloid formation *in vivo*^38^, under these *in vitro* conditions TTR presents a well-characterized non-nucleated aggregation reaction, devoid of any apparent lag phase, which in our hands reaches the maximal extent of aggregation around 55 h of incubation. TTR A108V and TTR A108I exhibited a dramatically reduced aggregation propensity, since the turbidity at the end of the reaction was 6 and 8-fold lower than the one recorded for TTR WT, respectively. For TTR aggregation, the development of turbidity and the formation of amyloid are known to correlate well. Accordingly, measurements of the extent of amyloid formation at the end point of the reaction using the amyloid-specific dye thioflavine-T (Fig. 4B), correlated with turbidimetric data, indicating that the two mutant proteins display a negligible aggregation propensity in these destabilizing conditions, a feature that was corroborated by inspecting the solutions aggregate content using transmission electronic microscopy. As shown in Fig. 4C, TTR A108V and TTR A108I solutions exhibit a low amount of aggregates when compared with TTR WT, for which the presence of prefibrilar aggegates displaying amyloid-like intermolecular β-sheet, according to their infrared spectra, could be observed (Fig. 4D, Fig. S1). For TTR A108V, a decreased tendency to fibrillation *in vitro* supports a decreased amyloidogenic potential *in vivo*.

**Figure 5:**
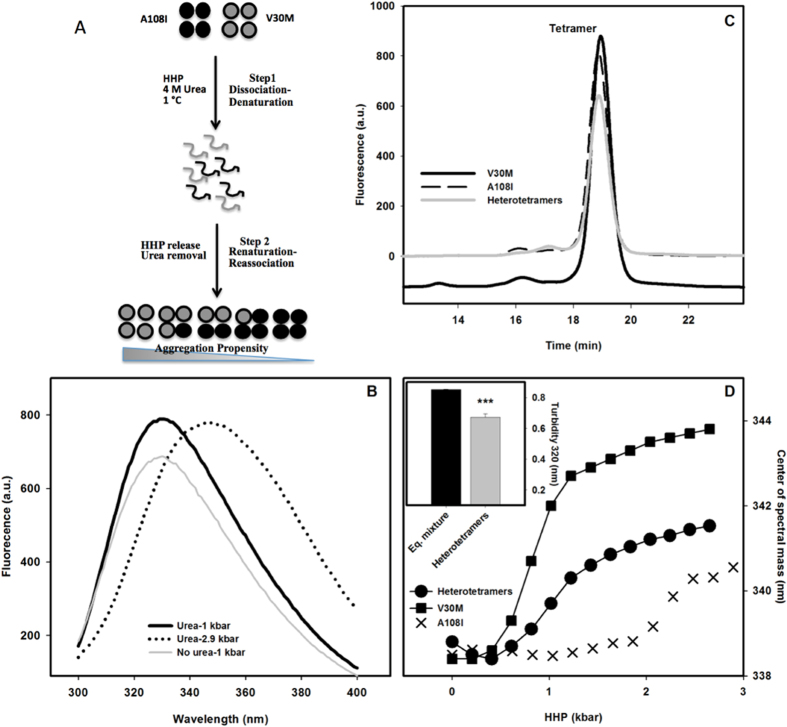
Formation and properties of A108I and V30M heterotetramers. Panel A shows a scheme of the protocol used to produce hybrid tetramers. Panel B shows Trp spectra emission of the mixture obtained at different moments of the assay, in which spectral shifts indicate the state of folding. The black continuous, black discontinuous and grey lines correspond to the spectra before application of HPP, after application of maximum HPP and upon refolding, respectively. Panel C shows the oligomeric state of the sample probed by SEC. The main peak on the chromatogram corresponds to the tetramer. Panel D shows the HHP titration assay. Trp fluorescence emission was used to report on the stability of the samples against increasing pressure. The results of an acidic induced aggregation assay for an equimolar mixture of non treated TTR A108V and TTR V30M and the hetroterameric mixture are shown in the inset.

We next addressed whether the incorporation of one or more subunits of TTR A108I could stabilize the pathogenic TTR V30M variant through the formation of heterotetramers, a phenomenon described before for TTR T119M^27^,^30^,^39^. In order to dissociate the stable TTR A108I tetramers, we used a combination of HHP, 4 M urea and low temperature^39^. Figure 5A shows a scheme of the assay. An equimolar mixture (15 μM V30M + 15 μM A108I) was submitted to HHP + urea and the Trp emission spectra was monitored. A red shift of 18 nm was observed when the sample was incubated in these conditions, indicating tetramer dissociation and monomer denaturation (Fig. 5B)^39^. HHP was released and the samples were dialyzed in order to remove the urea and allow the re-association of monomers, rendering a heterogeneous population of tetramers constituted by subunits of TTR A108I and TTR V30M in random ratio. We confirmed the refolding of TTR by monitoring the recovery of native Trp fluorescence (Fig. 5B). We also assesed the oligomerization state of the sample using SEC. Figure 5C shows that the heterotetramers produced by the protocol described above elute as a single peak around 19 min, exhibiting the same profile that the original TTR A108I and TTR V30M homotetramers. We performed next two different experiments to probe the conformational properties of the obtained heterotetramers: HHP titration (Fig. 5D) and an aggregation assay at pH 4.4 for 72 hs at 37 °C (Fig. 5D inset). HHP titration shows that the re-associated tetramers present an intermediate stability when compared with TTR V30M and A108I homotetramers (Fig. 5D). The aggregation assay shows that the heterotetramers were less aggregation prone than a fresh equimolar mixture of the two TTR variants (15 μM TTR V30M + 15 μM TTR A108I) (Fig. 5D inset).

### X-ray crystal structures of TTR A108V and TTR A108I variants

**Figure 6:**
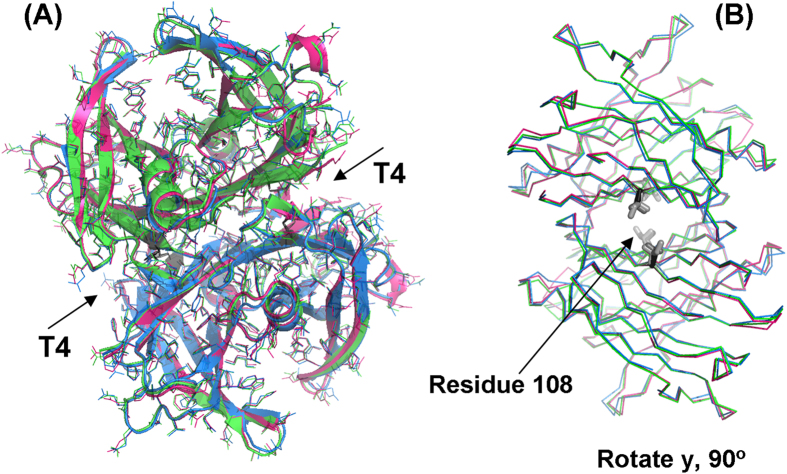
Comparison between the crystal structures of WT-TTR, TTR A108V and TTR A108I. (**A**) Superposition of TTR WT (pink), A108V (blue) and A108I (green) tetramers show overall rmsd for the superposed Cα atoms are 0.40 Å and 0.38 Å, respectively. (**B**) Rotation about y-axis makes possible to see that side-chain of residues at position 108 (gray-scale sticks) extend more and more inside of T_4_ channels in order Ala, Val and Ile108 variants.

Despite FoldX models predicted well the experimentally determined relative changes in protein stability, they only constitute an approximation to the real protein structures. To dissect unambiguously the molecular details accounting for the higher stability of the natural and designed TTR variants, we solved the crystal structures of both TTR A108V and TTR A108I homotetramers at 1.3 and 1.4 Å resolution, respectively. The structures are virtually identical to that of TTR WT, with overall rmsd values for the Cα of 0.40 Å for TTR A108V and 0.38 Å for TTR A108I (Fig. 6A). A rotation of 90° about y-axis shows that the side chains of residue 108 are projected inside the T_4_ binding channel at the tetrameric interface (Fig. 6B). The crystallographic parameters for both variants are shown in Table S1. The complete polypeptide chains could be traced with the exception of the terminal residues, 1 to 9 and 126 and 127, which were disordered and not defined in the electron density. Similar disorder regions have been reported in other TTR structures^26^,^40^. Interestingly, residues 36 to 40 and 98 to 104 in both chains, which belong to BC and FG loops, respectively, present well-defined electron densities and could be refined to moderate temperature factors, indicating a reduced mobility in comparison to the same loops in TTR WT and other TTR mutant structures, including the non-amyloidogenic TTR T119M, where they have been shown to display high flexibility^41^.

**Figure 7:**
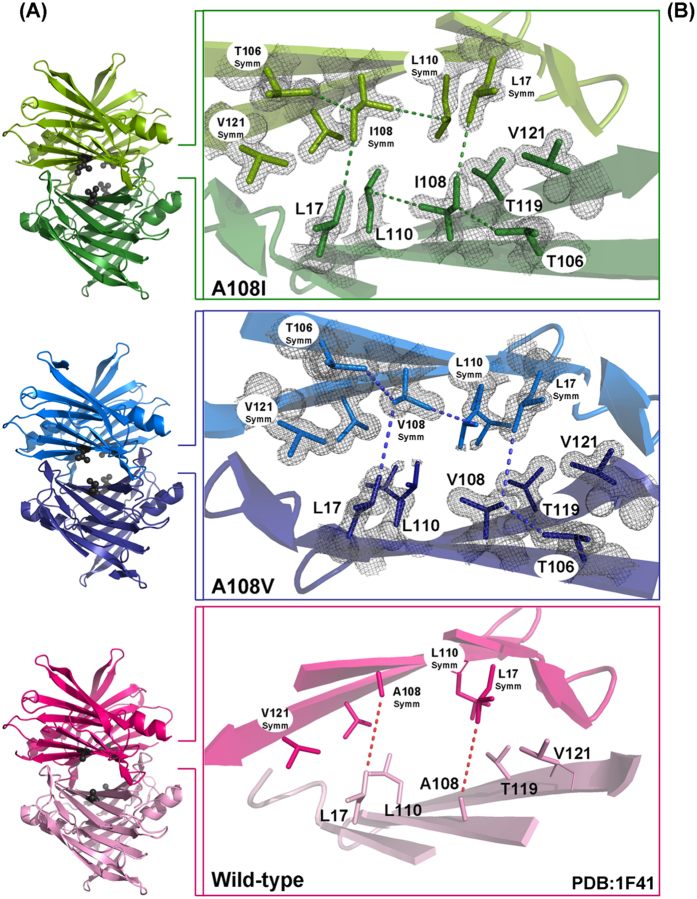
Tetrameric interfaces of the TTR WT and, A108V and A108I variants. (**A**) Cartoon diagrams of the TTR homotetramers showing the spatial orientation of residue A/V/I108 (black spheres) inside of the ligand binding pockets. (**B**) A glimpse in the thyroxine binding channel showing the new interactions formed after mutations that strengthen the dimer-dimer packing of these variants. Side-chains of the residues Leu17, Ala/Val/Leu108, Leu110, Thr119, Val121, important for protein stability, are shown in stick representation. The electron density maps are contoured at 1.5σ. Images were drawn with PyMOL.

Regarding to the tetrameric assemblies, the major finding on the TTR A108V and TTR A108I structures is a significant reduction of the volume of the ligand-binding hydrophobic cavity and the formation of a few novel interactions, namely between side chains of Val/Ile108 and Leu110 (3.80 Å), between Val/Ile108 and Thr106 (3.95 Å) inside the same monomer, and between Ile108 and Leu17^*Symm*^ (3.58 Å) (see Table S2). The effect of these novel interactions are amplified 4-fold across the AC/BD interface by the presence of the TTR tetrameric assembly. In addition to the formation of these novel interactions, it is remarkable the reduction of the hydrophobic cavity that binds T_4_ and other hydrophobic compounds, which is partially occupied in our TTR variants by the aliphatic side chains of Val108 or Ile108, leading to formation of a much closer AC/BD interface in the tetrameric assembly. These different interfaces in the TTR variants result in the decrease of the distance between the methyl groups of Cδ1 of Leu17^*Symm*^ with Cβ2 of Ala108 (at 7.80 Å distance), with Cγ2 of Val108 (at 4.25 Å distance), and with Cδ1 of Ile108 (at 3.58 Å distance) (Fig. 7).

Likewise, whereas the dimer interface area between AB and CD remains unvariable (Table 1), there is a significant increase in the AC/BD dimer-dimer contact area in the tetrameric assembly, with a relative increase of around 7% and 13% for the TTR A108V and TTR A108I mutant variants, respectively (Table 1). Therefore, structural comparison with T_4_-TTR and other ligand-bound TTR structures indicate that longer aliphatic side chains emanating from Ala108 position, such as our Val108 and Ile108 variants, would occupy a similar location as the T_4_-hormone. It is quite plausible that these mutant variants mimic the stabilizing effect produced by the filling up of the hydrophobic pocket by T_4_ or other hydrophobic ligands, such as the approved and in trial compounds for the treatment of TTR amyloidosis tolcalpone and tafamidis^26^,^42^.

### T_4_ binding to TTR A108V and TTR A108I

**Figure 8:**
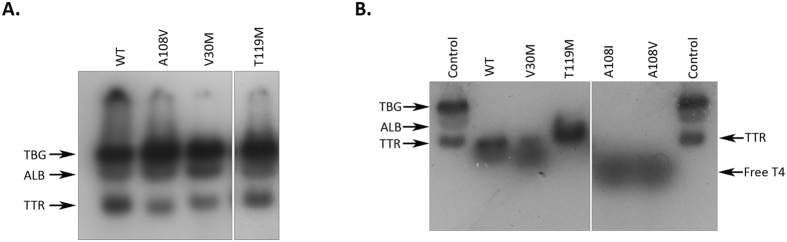
Analysis of T_4_ binding to plasma TTR (**A**) and recombinant TTR (**B**) by gel electrophoresis. The samples were incubated with ^125^I-T_4_ and the proteins were separated by native PAGE. Migration of TTR and other T_4_ binding proteins is indicated (TBG- thyroxine binding globulin; ALB- Albumin and TTR- Transthyretin). (**A**) Analysis of human plasma from heterozygote carriers of TTR variants TTR A108V, TTR V30M or TTR T119M and control plasma (WT) and (**B**) Analysis of isolated recombinant TTR variants, namely, V30M, A108, A108I, T119M and the wild type (WT) protein. Control plasma (control) was used as reference for migration. All samples were run in the same gel though not sequentially. The place where the gel was trimmed is indicated.

In TTR WT, the residue Ala 108 forms part of all the T_4_-Halogen-Binding Pockets: HBP1 and 1′ (Met13, Lys15, Leu17, Thr106, Ala108 and Val121), HBP 2 and 2′ (Leu17, Ala108, Ala109 and Leu110) and HBP 3 and 3′ (Ala108, Leu110, Ser117 and Thr119). This evidence, together with the fact that all the novel contacts established by the mutant Val108 and Ile108 side-chains involve residues in the hormone binding channel, suggested that the binding of T_4_ to TTR A108V and TTR A108I might be somehow, impeded. Therefore, we assayed the ability of T_4_ to bind to TTR in the plasma of the heterozygote subject carrying the A108V mutation. Plasma was incubated with [^125^I]-labelled T_4_, followed by separation of T_4_ binding proteins using native gel electrophoresis and T_4_ binding proteins visualization by autoradiography. The intensity of the bands corresponding to TTR in the different plasma samples was determined by densitometry. T_4_ binding to TTR in the plasma of controls (N) and from heterozygotes carrying the T119M or the V30M mutations, were also analysed as controls. TTR T119M and TTR V30M have been shown to display a higher and lower T_4_ affinity than TTR WT, respectively^28^. The intensity of the TTR band in plasma from the TTR A108V carrier was lower than the corresponding band in plasmas from control (N) and from the TTR T119M carrier resembling that of the TTR V30M carrier, indicating that, despite being highly stabilizing, the A108V mutation likely reduces the affinity for T_4_ (Fig. 8A).

In order to attribute the observed impact on T_4_ binding specifically to the presence of Val in position 108, the same assay was repeated with the isolated recombinant protein, as well as with the designed TTR A108I mutant, using recombinant TTR V30M and TTR T119M and TTR WT as controls. TTR A108V and TTR A108I, did not present any radioactive band corresponding to the migration of native TTR in gel electrophoresis after incubation with ^125^I-T_4_, demonstrating that these recombinant variants exhibit little affinity to bind T_4_, similar to what happens with TTR V30M and contrary to what is observed in TTR WT and TTR T119M (Fig. 8B).

**Figure 9:**
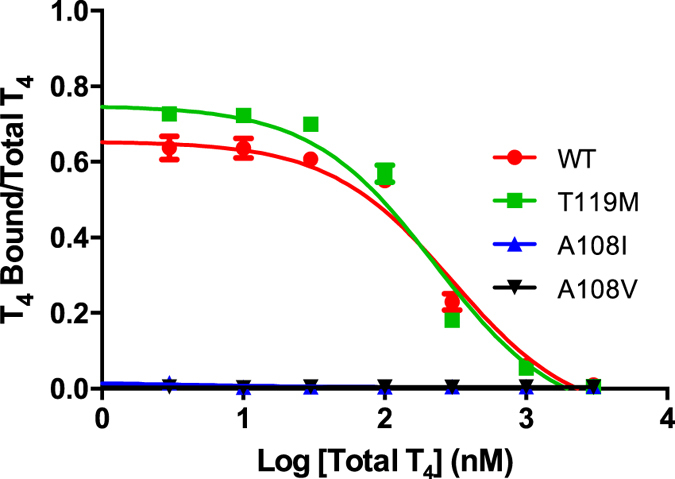
Competition curves of T_4_ for the binding to TTR variants obtained by gel filtration chromatography assay. The TTR variants tested were TTR WT, TTR T119M, TTR A108V and TTR A108I.

In order to attain a more precise characterization of T_4_ binding properties, a competition assay using gel filtration chromatography, was performed. In this assay, a constant amount of each TTR variant was incubated with a small amount of [^125^I]T_4_ and with increasing concentrations of non-labelled T_4_ as competitor. T_4_ bound to TTR was separated from the unbound molecule in the size exclusion column. The results were expressed as percentage of binding against logarithm of the concentration of non-labelled T_4_. The obtained competition curves are presented in Fig. 9. In good agreement with the electrophoretic data, the assay clearly demonstrates that the 108 mutant TTR variants do not exhibit T_4_ binding affinity as no competition was observed, in contrast to what happens for TTR WT and TTR T119M, that were tested in parallel.

## Discussion

The intrinsic aggregation propensity of TTR WT causes that as much as 25% of the population above 80 years old suffers from SSA^3^. Pathogenic mutations associated with inherited TTR amyloidosis make the protein more susceptible to aggregation. To date, it is still not completely clear how TTR amyloid formation occurs under physiological conditions, but the most accepted hypothesis assumes that mutations in TTR impact the structural equilibrium of the protein, destabilizing the tetramer in favour of dissociated intermediates, which exhibit amyloidogenic potential. The age of onset and the penetrance of the disease depend on the specific mutation. As a general trend, the more destabilizing the mutation is, the more pronounced the amyloidogenicity of the protein variant. Mutations directly or indirectly affecting the stability of the AC/BD dimer-dimer interface tend to promote exacerbated protein aggregation and severe disease phenotypes, highlighting the crucial role played by the native quaternary structure of TTR at preventing protein deposition. It is worth to point out here that an alternative mechano-enzymatic cleavage mechanism has been recently proposed to underlie transthyretin amyloidogenesis *in vivo*^43^. According to this new view, the combined effect of proteolysis and shearing would result in the release of a highly amyloidogenic 49–127 truncated protomer, usually found in *ex vivo* deposits. Destabilized TTR variants would be more susceptible to proteolysis than TTR WT^43^.

The number of non-amyloidogenic mutations identified so far for TTR is small^44^, which is consistent with the idea that most sporadic mutations in proteins have a destabilizing effect^45^. Some mutations, such as G6S^46^ or H90N^47^ have a negligible impact on TTR stability and are considered non-pathogenic, whereas others like R104H^48^ or T119M^30^ have been shown to act as trans-suppressors. Trans-suppressor mutations render TTR more stable against dissociation and aggregation, reducing the symptoms of the disease in heterozygotes carrying additional aggressive mutations in the other allele. The trans-suppressor effects of R104H and T119M respond to different mechanisms^48^. The quaternary structure of R104H is thermodynamically stabilised, whereas the tetrameric native state of T119M exhibits a high kinetic stability. The stabilizing effect of T119M mutation is much higher than that of R104H, which only modestly protects against aggregation. Thr119 protrudes directly into the T_4_ binding site and the high kinetic stabilization provided by its mutation into Met owes to the novel contacts that this hydrophobic residue establishes or induces between amino acids belonging to different dimers, which strengthen the interaction between dimers at the T_4_ binding pocket. This is the same principle exploited by hundreds of small compounds that by binding to and filling the two T_4_ binding sites stabilize the native state of TTR over the dissociative transition state. Thus, reinforcing the contacts at the quaternary dimer-dimer interface appears as a requirement to impose kinetic stability on the entire TTR tetramer. It is worth to emphasize here that T119M TTR is also totally resistant to proteolytic cleavage under shearing^43^.

Here we report on a novel mutation affecting residue Ala108 that as Thr119 is located in the T_4_-binding cavity. Mutations of the adjacent Ala 109 residue to Thr^49^ or Val^50^ are non-amyloidogenic, whereas mutation to Ser is pathogenic^51^; however, Ala 109 does not face the T_4_ binding cavity. The subject bearing the mutation A108V in heterozygoty presented a mild, non-progressive, sensory neuropathy without any dysautonomic symptom or sign, a course that is not typical of a TTR-related polyneuropathy. Since it was not possible to follow-up the patient for more than 3 years, and there was almost no progression of disease, and no family history was available, the atypical picture suggests an idiopathic neuropathy or, more interesting, that the novel mutation is associated with a very benign expression of TTR-associated disease. Initial analysis of the stability of the TTR in the blood of the subject as well as computational predictions already suggested that the mutation could be overstabilizing, a fact that was confirmed by characterizing the stability features of the recombinant protein, which only begins to unfold upon incubation in 6 M urea for 96 h. Globally, the A108V mutation adds four additional methylene groups, two at each of the T_4_-binding sites at the dimer-dimer interface. This is important, because experiments with small molecules demonstrate that their binding to the first and second T_4_-binding sites additively increase the activation barrier associated with tetramer dissociation. The generated structural model suggested that the interface would still admit four additional methylenes in the form of an A108I mutation, without introducing steric clashes that might result in strain at the interface. In good agreement with the prediction, the recombinant TTR A108I protein turned to be significantly more stable than TTR A108V, maintaining its native tetrameric structure intact upon incubation on 8 M urea for 96 h. Their high stabilities in front of urea denaturation at equilibrium and, specially, the very low dissociation rates they exhibit under highly denaturing conditions, are consistent with the existence of a high kinetic barrier for the dissociation TTR A108V and TTR A108I homotetramers. HHP causes denaturation of proteins by promoting the entrance of water molecules into the protein cavities, facilitating the population of partially folded states^52^, which in TTR, affects the dissociation of the tetramer. The occupation of the T_4_-binding sites by the additional methylene groups of Val and Ile makes these variants, specially TTR A108I, resistant to pressure. This effect may respond either to a stabilization of the ground state of the protein or to a destabilization of the dissociative transition state. In any case, it results in a dramatic kinetic stabilization for these proteins, which is translated in almost a complete suppression of their aggregation under acidic conditions. Interestingly enough, no mutation has previously been found in position 108 of TTR. Thus, our data provide yet another piece of the puzzle in elucidating the correlation between mutations and aggregation propensity in the context of TTR amyloidoses.

The almost 200 solved X-ray structures of TTR WT and its variants have not revealed major conformational changes that could explain their differential amyloidogenicity^53^,^54^. They are rather small differences at specific positions that account for changes in protein stability^19^,^41^,^55^. The crystal structures reported here show that the dimer-dimer contact area is more extensive in TTR A108V and in TTR A108I than in TTR WT. Thus, the quaternary structure of the mutant variants is assembled by additional new inter-subunit contacts that might account for their resistance to dissociation. A synthetic TTR L110A variant has been shown to exhibit little resistance to dissociation^14^. This destabilization has been attributed to the fact that Leu110 is in the dimer-dimer interface and the substitution will reduce the contact area. Conversely, in TTR A108V and TTR A108I, Val/Ile108 side chains interact with Leu110 and Thr106 inside the same monomer, as well as with Leu17^*Symm*^ at AC/BD interface, in the case of TTR A108I. These contacts, absent or weak in TTR WT, repack the hydrophobic core of the T_4_-binding cavity stabilizing the tetramer. The side chain of Ala108 is part of the three TTR HBP sites and occupies a central position in the cavity, whereas Thr119 is only involved in HBP3 and located in one of the extreme of the funnel. This explains why an increase in the hydrophobic volume of the residue in position 108 results in a higher stabilizing effect than when the same type of change occurs in position 119. The high stability of the TTR A108V and TTR A108I structures allow the residues in loops BC and FG to have well-defined electron densities and to refine to moderate temperature factors, suggesting higher rigidity as compared with TTR WT and other TTR variant structures, including TTR T119M. This is important, because a high mobility of the BC loop has been observed for the artificial and highly amyloidogenic TTR G53S/E54D/L55S triple mutant^56^. Indeed, all the mutations described in this loop are linked to disease.

It is worth to emphasize, that trans-suppressor mutations of TTR are of considerable interest, because they can be potentially employed to treat TTR-related amyloid diseases using liver targeted gene therapy. In fact, T119M gene therapy in a V30M transgenic mice showed a prophylactic effect on reducing TTR deposition. TTR A108I, is perhaps the most stable mutant attained so far with a single amino acid change not involving covalent cross-linking. The high stability in front of HPP denaturation and the reduced aggregation of V30M/A108I heterotetramers, relative to V30M homotetramers, suggests a potential application in therapy for this designed variant, although additional experiments are required to confirm this extent.

The fact that TTR A108V and TTR A108I display a low binding affinity for T_4,_ should not be an impediment for their involvement in putative prophylactic therapies, since only a small fraction of TTR circulates in a bound state, thyroxine-binding globulin (TBG) being the main T_4_ binding protein in the blood. Indeed, small molecules designed to impair TTR aggregation exert their stabilizing effect by binding with high affinity to the T_4_-binding site, thus competing the binding of the hormone. The novel contacts established by the Val and Ile side chains inside the T_4_-binding cavity should turn to be useful to engineer barrier heights in kinetic stabilizers by functionalizing them with chemical groups that by mimicking those interactions would strengthen the contacts at the dimer-dimer interface to the level observed in these two highly stable, non-amyloidogenic mutants.

## Material and Methods

### Case report

A 73-year-old healthy woman reported a 12-months history of insidious onset and mild progression of symmetrical numbness of the feet with painful dysaesthesia. Pain sensation was slightly reduced below ankles. Vibration and position sensation were normal. Ankle reflexes were weak, otherwise tendon reflexes were normal. Ataxia was absent and muscle strength was normal. There was no evidence of autonomic nerve or pyramidal tract dysfunction. There was no previous history of diabetes, alcohol intake or drug toxicity. Family history was negative for polyneuropathy or other neurological disorders. Nerve conduction studies confirmed mild sensory neuropathy, with absent sural nerve action potential on both sides, but normal sensory potentials in upper limbs (radial and median, bilaterally) and unremarkable motor nerve conduction velocities and F-waves (median and peroneus, bilaterally). The following blood tests were normal or negative: full blood count, glycaemia, liver and renal function tests, thyroid hormone and vitamin B12 levels, serum immunoglobulins, autoantibody testing for anti-nuclear, extractable nuclear antigen and anti-neutrophil cytoplasmic antibodies, hepatitis C and HIV-1 serology. Investigation of TTR V30M mutation was negative. Over the following three years the patient was observed every 6-months and a comparative nerve conduction study was repeated every year, without any evidence of disease progression. Following this period the patient was lost for follow-up. Blood samples were collected according to standards established by the latest revision of the Declaration of Helsinki. All methods were performed in accordance with the relevant guidelines and regulations. Informed consent was obtained from all donors in this study, including the propositus. They provided written permission for genetic tests relevant to determine the cause of their clinical condition and for research in the disease, for diagnostic purposes investigations do not require Ethics Committee approval in our centers.

### Plasma TTR immunoprecipitation and peptide mass spectra

Briefly, 25 μL of serum were incubated with anti-human TTR antibody and kept overnight at 4 °C with stirring. Next, protein A Sepharose beads were added and allowed to mix for 1 h at room temperature with agitation. The beads were washed and the complex eluted with SDS-loading buffer. Finally, samples were fractioned on a 15% polyacrylamide gel under denaturing conditions (SDS-PAGE) and the gel was stained following the Coomassie Colloidal Blue protocol. The TTR monomeric band was excised from the gel and in-gel digestions were performed, after reduction and alkylation, by adding modified porcine trypsin (Promega). Peptide mass spectra were acquired using Applied Biosystems 4800 *Plus* MALDI-TOF/TOF MS equipment.

### DNA amplification and sequence analysis

DNA was extracted from peripheral blood cells and exon 4 from the propositus was amplified with primers flanking the coding region [forward 5′-CACGTTTTTCGGGCTCTGGTGG-3′ and reverse 5′-CTTCCTGCTCCCTACCCTAAAG-3′ (nucleotides 7222–7425), which amplified a fragment of 203 base pairs. PCR products were purified using a High Pure PCR Purification Kit (Roche, Basel, Switzerland) and sequenced with a BigDye Terminator v3.1 Cycle Sequencing Kit (Applied Biosystems, Foster City, CA, USA). Sequences were analyzed using ChromasPro and Ridom TraceEdit.

### *In Silico* Stability Predictions

We built two different models (TTR A108V and TTR A108I) by using FoldX (http://foldx.crg.es/) command Buildmodel with the original TTR WT structure as deposited on the PDB 1F41; each model was generated with five runs, and they converged well. The resulting structures were used to calculate the ΔΔGs values presented in the Results section. The command Stability was used to calculate the ΔΔGs of unfolding, between variants. The energies are an automatic output in FoldX, and the changes in native stability upon mutation were estimated as the difference between the energy of the wild type protein and that of the mutant protein (ΔΔG = ΔGmut − ΔGwt). ΔΔGs values above 1.6 kcal/mol should significantly affect variant stability, because they correspond to twice the intrinsic standard deviation of FoldX.

### Recombinant TTR expression and purification

Recombinant WT, TTR A108V and TTR A108I were prepared as detailed in ref. 57.

### TTR aggregation assay

3.5 μM of native proteins were incubated under quiescent conditions in 200 mM sodium acetate, 100 mM KCl, pH 4.4, at 37 °C. After 72 h, samples were vortexed, and their turbidity (330 nm) was monitored along time on a UVI-Vis Carry spectrophotometer (Shimadzu).

### TTR stability assays

#### *In vitro* assay of Trp fluorescence

TTR WT (1.8 μM) was incubated with different urea volumes to attain a range of final concentrations (0 M to 9 M) and samples were incubated for 96 h at RT. Fluorescence measurements were performed on a Jasco FP-8200 Spectrofluorometer. Trp fluorescence was obtained by exciting the samples at 280 nm. Emission was collected from 300 to 400 nm. The fluorescence intensity ratio 355/335 nm was plotted against urea concentration as a sensor of folded protein, which for TTR in urea is equivalent to tetramer integrity^15^. In another setting, TTR WT and mutants (1.8 μM in PBS) were incubated at 8 M urea and fluorescence measured along time at RT. The fraction of unfolded TTR was measured and calculated as above. TTR variants stability was also assessed by HHP. Experiments were performed at 1 μM of each mutant at pH 7.5 and 1 °C. The high-pressure cell equipped with optical windows was purchased from ISS (Champaign, IL). Fluorescence spectra were recorded on an ISS K2 spectrofluorometer. Pressure was increased in steps of 200 bar. At each step the sample was allowed to equilibrate for 15 min before making measurements. Tryptophan emission spectra were obtained by setting the excitation at 280 nm and collecting the emission in the 300- to 400-nm range. Shifts in the center of spectral mass were used to follow denaturation.

#### Assay of TTR tetrameric stability by Isoelectric Focusing (IEF)

Stability of recombinant TTR or TTR in whole plasma was assessed by isoelectric focusing (IEF) in semi-dissociating conditions as previously described^24^. In brief, 30 μL of human plasma were applied to native PAGE for the separation of plasma proteins and the gel band containing TTR was excised and applied to an IEF gel. IEF was carried out in semi-denaturing conditions (4 M urea), containing 5% (v/v) ampholytes pH 4–6.5 (GE Healthcare), at 1200 V for 6 hours. Proteins were stained with Coomassie Blue, the gels were scanned and subjected to densitometry using the ImageQuant program. The results were expressed as the ratio of TTR tetramer over total TTR. The data were analysed by Student’s t-test using Microsoft Excel to establish significant changes.

#### Transmission Electron Microscopy

Aggregated samples were diluted in Mili-Q water to 1 μM. 5 μl of the solution were adsorbed onto carbon-coated copper grids for 5 minutes and blotted to remove excess material. Uranyl acetate (0.5% w/v) was used for negative staining. Samples were dried on air for 5 minutes. Grids were exhaustively scanned with a Hitachi H-7000 transmission electron microscope operating at a voltage of 75 kV.

#### Thioflavin T (Th-T) Binding Assay

Th-T binding was used to probe amyloid formation on the samples. 10 μl of Aggregated samples were added a Th-T solution in 5 mM sodium phosphate, pH 8, resulting in a final Th-T concentration of 20 μM. Excitation was set to 450 nm, whereas emission was collected from 450 to 600 nm. Five individual scans were averaged for each measurement using Jasco 8200 spectrofluorometer. The intensity of the spectra at 482 nm was used as an indicator of the extent of amyloid material.

#### Fourier transform infrared spectroscopy

ATR-FTIR analysis of the aggregated samples was carried out using a Bruker Tensor 27 FTIR spectrometer (Bruker Optics) with a Golden Gate MKII ATR accessory. 5 μL of sample were placed in the centre of the diamond and dried with N2. Each spectrum consisted of 16 scan accumulations measured at a spectral resolution of 2 cm^−1^ in a wavelength range between 1700 and 1500 cm^-1^.

#### Production of Heterotetramers by HHP Treatment

An equimolar mixture of TTR V30M and TTR A108I (15 μM each) was subjected to HHP (29 kbar) in the presence of 4 M urea in PBS at 1 °C for 60 min to allow for the dissociation of TTR tetramers. Pressure was released and the mixture was dialyzed for 24 h at 4 °C against PBS to remove residual urea and to allow for re-association and subunit exchange^39^. To probe the oligomeric state of the sample, high performance liquid chromatography was performed in a Superdex 75 10/300 GL column (GE healthcare Life Sciences) at room temperature using an HPLC system at a flow rate used was of 0.5 ml/min. (Shimadzu SPD-10A). The system was equilibrated in PBS. Sample elution was monitored by Trp fluorescence at 320 nm.

#### Crystallography and Structure Determination

Crystals of TTR A108V and TTR A108I were obtained at 18 °C by hanging-drop vapor diffusion methods after purification and concentration. The reservoir solution contained between 15–25% PEG 400, 200 mM calcium chloride, 100 mM HEPES, pH 7.5. Single crystals appeared after three days from equal volumes of protein solution (10 mg/mL in 50 mM Tris-HCl pH 8.0, 100 mM KCl, 1 mM EDTA) and reservoir solution. Crystals were directly flash-frozen in liquid nitrogen prior to diffraction analysis. Diffraction data were recorded from cryo-cooled crystals (100 K) at the ALBA synchrotron in Barcelona (BL13-XALOC beamline). Data were integrated and merged using XDS^58^ and scaled, reduced and further analyzed using CCP4 (Table S1). The structures of TTR variants were determined from the X-ray data by molecular replacement using a previous TTR structure (PDB 1F41) as a model using the program Phaser^59^. Model refinement and rebuilding were performed with PHENIX^60^,^61^ and Coot^61^. Refinement and data statistics are provided in Table S1, structural representations were prepared with PyMol^62^. Those structures are deposited at PDB codes 5FW6 and 5FO2, respectively.

### Thyroxine binding to TTR variants

#### Assay by native polyacrylamide gel electrophoresis (PAGE)

Thyroxine (T_4_) binding to TTR was assayed by incubation of plasma samples or isolated recombinant TTR, or TTR variants, with radiolabeled thyroxine ([^125^I]T_4_) followed by protein separation by native polyacrylamide gel electrophoresis (PAGE) and detection of T_4_ binding proteins after autoradiography. In more detail, 5 μL of plasma or 10 μg of recombinant TTR were incubated with 0.25 μL of [^125^I]T_4_ (specific radioactivity 1250 μCi/μg; concentration 320 μCi/mL; Perkin Elmer, Boston, MA, U.S.A.) for 1 hour at room temperature. Samples were submitted to electrophoresis for proteins separation by native PAGE^24^. After electrophoresis the gels were dried, and exposed to autoradiography film. The film was scanned and the intensity of the protein bands was quantified by densitometry.

#### Assay by gel filtration chromatography

T_4_ binding to isolated TTR was also studied using a competition assay by gel filtration chromatography. Relative affinity of T_4_ to bind to different isolated recombinant TTR variants was determined by gel filtration, as described previously, using a constant concentration of each TTR variant (30 nM) incubated with a tracer amount of [^125^I]T_4_ (approx. 50 000 cpm) and with increasing concentrations of non-labelled T_4_ (0–1 μM final concentration - 7 different concentrations) as competitor. T_4_ bound to TTR was separated from unbound by filtration through a P6DG gel filtration column (BioRad). The results were expressed as percentage of binding against logarithm concentration of non-labelled T_4_. Competition curves obtained were compared.

## Additional Information

**How to cite this article**: Sant’Anna, R. *et al*. Cavity filling mutations at the thyroxine-binding site dramatically increase transthyretin stability and prevent its aggregation. *Sci. Rep.* **7**, 44709; doi: 10.1038/srep44709 (2017).

**Publisher's note:** Springer Nature remains neutral with regard to jurisdictional claims in published maps and institutional affiliations.

## Supplementary Material

Supplementary Information

## Figures and Tables

**Table 1:** Interface area involved in the inter-subunit contacts of TTR WT, TTR A108V and TTR A108I.

	TTR WT	TTR A108V	TTR A108I
Dimer Interface Area (Å^2^)	1774	1731	1752
Tetramer Interface Area (Å^2^)	766	829	880

TTR WT, A108V and TTR A108I are deposited under PDB codes 1F41, 5FW6 and 5FO2. The calculations were performed using PDBSum web server.
