# Supplementary material for: Cavity filling mutations at the thyroxine-binding site dramatically increase transthyretin stability and prevent its aggregation
Source: Sci Rep. 2017 Mar 24;7:44709. doi: 10.1038/srep44709 (PMC5364509; doi:10.1038/srep44709)
Supplement: Supplementary Information [file srep44709-s1.pdf]

Supplementary information:

Cavity filling mutations at the thyroxine-binding site dramatically increase transthyretin stability and prevent its aggregation

Ricardo Sant'Anna[1,5], Maria Rosário Almeida [2,3,4], Nathalia Varejão[1,5], Pablo Gallego[1], Sebastian Esperante[1], Priscila Ferreira[5], Alda Pereira-Henriques[2,3], Fernando L. Palhano[5], Mamede de Carvalho[6], Debora Foguel[5], David Reverter[1], Maria João Saraiva[2,3] and Salvador Ventura[1]

[1]Institut de Biotecnologia i Biomedicina and Departament de Bioquímica i Biologia Molecular, Universitat Autònoma de Barcelona, Bellaterra, Spain.

[2] i3S – Instituto de Investigação e Inovação em Saúde da Universidade do Porto, Rua Alfredo Allen, 208, 4200 – 135 Porto, Portugal

[3] IBMC - Instituto de Biologia Molecular e Celular, Universidade do Porto, Rua Alfredo Allen, 208, 4200 – 135 Porto, Portugal

[4] ICBAS, Instituto de Ciências Biomédicas Abel Salazar, Universidade do Porto, Rua Jorge Viterbo Ferreira 228, 4050 – 313 Porto, Portugal

[5]Instituto de Bioquímica Médica Leopoldo de Meis, Federal University of Rio de Janeiro, Rio de Janeiro, Brazil

[6] Institute of Physiology-Instituto de Medicina Molecular, Faculty of Medicine, University of Lisbon, Portugal; Department Neurosciences, Hospital de Santa Maria-CHLN, Lisbon, Portugal.

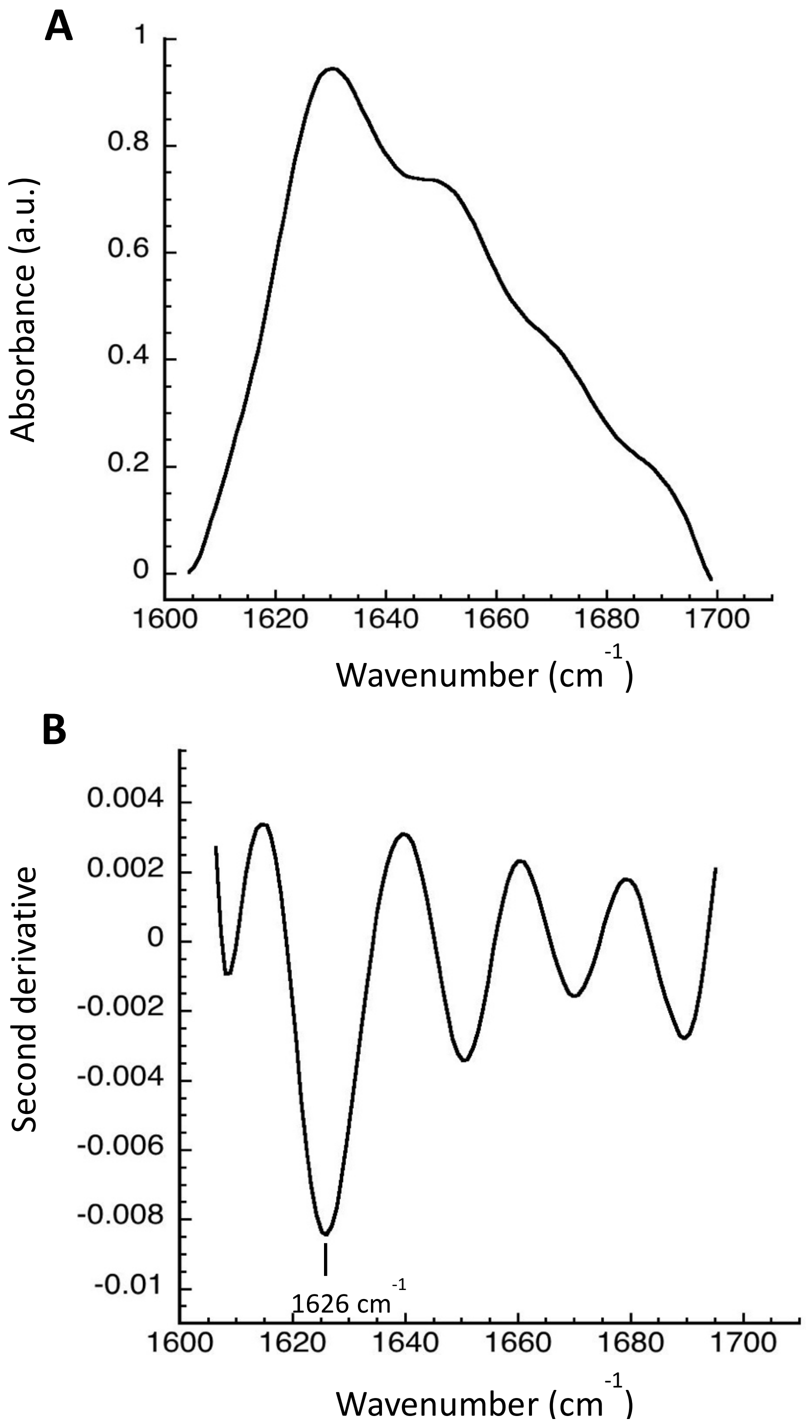

Figure S1. Secondary structure analysis of TTR WT aggregates. A) ATR-FTIR absorbance spectra in the Amide I region of the TTR WT aggregates shown in Fig. 4C. B) Second derivative of the absorbance spectra. The band correspondent to inter-molecular β-sheets is indicated.

Table S1 – Data collection and refinement statistics. Values in parentheses are for the last shell.

	TTR A108V	TTR A108I
**Data collection**		
Space group	$P2_122_1$	$P2_12_12$
Unit cell parameters (Å) a, b, c	43.69, 64.92, 85.03	85.25, 43.59, 64.92
Wavelength (nm)	0.97927	0.97935
Oscillation range (°)	0.25	0.25
Resolution range (Å)	64.92-1.30	85.25-1.44
Rmerge	0.035(0.50)	0.060(0.67)
(I/σ(I))	22.9(3.6)	14.6(2.2)
Completeness (%)	99.8(98.8)	98.0(93.3)
Multiplicity	6.5(6.2)	6.3(5.8)
**Structure refinement**		
Resolution range (Å)	64.92-1.30	64.9-1.44
No. of unique reflections	57155	42865
Rwork/Rfree (%)	17.12/20.29	18.52/21.51
No. of atoms		
Protein	1907	1816
Water molecules	178	272
Overall B factors (Å^2)	20.27	20.40
R.m.s. deviations		
Bonds (Å)	0.030	0.018
Angles (°)	2.40	1.75
**PDB code**	**5FW6**	**5FO2**

Table S2 – Interatomic contacts between A/V/I108 residues at the tetrameric interface of TTR crystal structures.

Monomers A---C	No-polar interactions (A)	TTR WT*	TTR A108V	TTR A108I
	Leu17 CG---CB Ala/Val/Ile 108	7.08	6.28	7.21
	Leu17 CD1---CB Ala/Val/Ile 108	5.58	4.77	5.70
	Leu 17 CD1---CG1 Val/Ile 108	---	4.90	5.09
	Leu 17 CD1---CG2 Val/Ile 108	---	**4.25**	5.96
	Leu17 CD1---CD1 Ile108	---	---	**3.58**
	Leu17 CD2---CG2 Val/Ile 108	---	6.15	8.25
	Leu17 CD2---CD1 Ile108	---	---	5.49

*Atomic coordinates deposited in the PDB under entry code 1F41.
